# Insomnia and its association with absenteeism: A cross-sectional study among Iranian nursing team

**DOI:** 10.5935/1984-0063.20200106

**Published:** 2021

**Authors:** Khosro Sadeghniiat-Haghighi, Arezu Najafi, Sahar Eftekhari, Samareh Tarkhan

**Affiliations:** 1 Tehran University of Medical Science, Occupational sleep research center - Tehran - Iran.; 2 Tehran University of Medical Science, Center for Research on Occupational Disease - Tehran - Iran.

**Keywords:** Insomnia, Healthcare provider, Nursing team, Absenteeism

## Abstract

**Objective:**

Given the potential impact of insomnia on nurses’ performance, it is assumed that insomnia is associated with their absence from work. The present study aimed to determine the insomnia status and its association with absenteeism among a selective group of Iranian healthcare providers.

**Material and Methods:**

This cross-sectional study was conducted on 304 healthcare providers working at Imam-Khomeini hospital complex in Tehran. The study population were assessed by insomnia severity index for characterization of insomnia symptoms. The data of absenteeism was collected from the employees’ attendance system of hospital’s nursing and staff department. The multivariable linear regression model used for predicting determinants of insomnia and absenteeism in nursing team.

**Results:**

Different degrees of insomnia was found in 79.9% of the study population, which 57.2% suffered from mild insomnia, 21.4% from moderate insomnia, and 1.3% from severe insomnia. The prevalence of insomnia was significantly higher in persons who were absent from their workplace frequently, or left because of illness. The mean days for total absenteeism in healthcare workers with moderate to severe insomnia was significantly higher than others with mild and no insomnia. In multivariate analysis, having night shifts and the severity of insomnia could predict absenteeism in studied population.

**Discussion:**

A majority of healthcare workers suffer from insomnia that may lead to their work absenteeism and decreased performance. Proper administrative and individuals for management of sleep problems is required to avoid long hours of absenteeism among nursing team.

## INTRODUCTION

Sleep and rest are one of the basic human needs and are in the series of Maslow’s needs in the line of physiological requirements^1^. Other physiological functions of the body may also change when the sleep-wake cycle is disrupted. Sleep deprivation can lead to fatigue, anorexia nervosa, loss of concentration, exacerbation of illness, and physical discomfort^2^. As a result of sleep deprivation, the hormone adrenaline enters the bloodstream more and more, so the person feels tired and depressed, and his or her concentration also decreases^3^. Common sleep-disorders-related complications include increased cardiovascular disease, gastrointestinal disorders, mental disorders such as depression and anxiety, decreased proper functioning, increased drowsiness, and the occurrence of occupational and non-occupational errors, and even the tendency to take medication^4, 5^.

Nurses are the largest professional group in the healthcare system including 40% of all hospital staff and 55% of the total staff costs^6^. Therefore, they play a significant role in the healthcare system. Nurses are one of the most prominent groups in shift work, and the occurrence of insomnia and sleep disorders not only affects their health but also endangers the health of patients under their supervision^7, 8^. Sleep disorder is one of the most important factors that reduce the quality of nurses’ performance and cause errors in the treatment of patients and can cause irreparable damage.

Absence from work or absenteeism is one of the indicators affected by sleep status and faces the individuals with many problems, especially among nursing and other healthcare providers. Absenteeism is defined as “allocating time to non-work activities when the person is expected to be working”^9^. Of course, absenteeism not only means the physical absence of individuals, but also the absence of psychological presence or the ineffective presence and inefficiency of individuals. There are different types of absenteeism, including short-term absenteeism, long-term medical absenteeism, unauthorized or persistent delays, and authorized absenteeism^10^. Absenteeism is also seen as a sign of resignation, as individuals try to avoid negative working conditions by doing negative things such as absenteeism^11^.

Absence from work in the nursing staff causes other people to bear the extra burden, which in turn causes fatigue and job dissatisfaction among them^12^. In addition, absenteeism reduces the quality and quantity of nursing care and ultimately reduces community health^13, 14^. The high rate of absenteeism also undermines the minimum care available to patients and clients and threatens the health of society as a whole. The lack of each nurse in his or her position in terms of providing medical services has had an adverse effect on the entire scientific management system and has disrupted the healthcare system, the effect of which is directly on the patients’ health^15^. Long and frequent absences from work will eventually lead to leaving work and career. Although nurses’ absenteeism may have some benefits such as the withdrawal of dissatisfied, demoralized, or sick workers who are unable to work, and is primarily economically beneficial to the organization, if material costs and management problems are considered which is a very small advantage^16^. This will impose a lot of material costs on hiring and retaining nursing services, spending more time and money to train people to replace employees who are out of service or absent from work.

Given the potential impact of insomnia on nurses’ performance, it is assumed that insomnia in this group of healthcare providers is associated with their absence from work. Thus, due to limited information in this regard we aimed to assess the insomnia characteristics among nursing team and to determine whether there is an association between insomnia and work absenteeism.

## MATERIAL AND METHODS

### Study population

This cross-sectional study was conducted on 304 healthcare provider including nurses, head nurses, paramedics and operating room technician, working at Imam Hospital complex, in Tehran, between 2017 and 2019. Stratified sampling and simple random sampling techniques were used to determine the study population. The employees were stratified into 3 major ward including internal ward, surgical ward and intensive care wards (CCU, ICU, and NICU) as well as some minor wards entitled “other wards”. The participants were selected from the list of nursing team of each wards using simple random sampling.

A total of 370 questionnaires were distributed between the nursing team of different wards and 304 questionnaires were completed and analyzed. After approval the implementation stages of the project in the ethics committee of the Tehran University of Medical Sciences and providing full explanations to the nurses about the objectives of the study, the investigation was started. Initial information from the nurses, including data on demographic characteristics and their job descriptions was collected through interviews. The data of absenteeism was collected from the employees’ attendance system of hospital’s nursing and staff department. The following definitions were used for calculating this data:

- Unauthorized absence is when the employee does not come to work and does not contact the employer or gives no reason for his/her absence;
- Sick absence is paid time off from work that workers can use to stay home to address their health needs without losing pay;
- Vacation means the time allotted by the employer for the employees to not be present at work;
- Total absence is total absence days from work (for authorized and unauthorized reasons).

### Study measurements

In a study by Sadeghniiat et al. (2015)^17^, the validity and reliability of the Persian version of the questionnaire is approved. For assessing the severity of insomnia, the insomnia severity index was employed. The insomnia severity index (ISI) is a 7-item self-report questionnaire assessing the nature, severity, and impact of insomnia. The usual recall period is the “last month” and the dimensions evaluated are: severity of sleep onset, sleep maintenance, and early morning awakening problems, sleep dissatisfaction, interference of sleep difficulties with daytime functioning, noticeability of sleep problems by others, and distress caused by the sleep difficulties. A 5-point Likert scale is used to rate each item (0 = no problem; 4 = very severe problem), yielding a total score ranging from 0 to 28. The total score is interpreted as follows: absence of insomnia (0-7); sub-threshold insomnia (8-14); moderate insomnia (15-21); and severe insomnia (22-28)^18^. Yazdi et al. (2012)^19^ demonstrated high reliability and validity of the ISI by the Cronbach’s alpha coefficient above 0.8 and intra-class correlation coefficient above 0.7. The absence data of the nursing team included unauthorized absence, sick absence as well as vacation days and hours in a time period of 30 months.

### Statistical analysis

The results are presented as mean ± standard deviation (SD) for quantitative variables and were summarized by absolute frequencies and percentages for categorical variables. Quantitative variables were also compared with t test or Mann-Whitney U test and one-way ANOVA. For the statistical analysis, the statistical software SPSS version 22 for windows (SPSS Inc., Chicago, IL, USA) was used. The *p* values of 0.05 or less were considered statistically significant; *p* for trend was used to test a linear trend of absenteeism rate between the categories of insomnia grading. The multivariable linear regression model used for predicting determinants of insomnia and absenteeism in nursing team.

## RESULTS

In total, 304 healthcare workers were assessed, with the average age of 35±8.38 years. The mean times for unauthorized absence, vacation, and sick absence were 0.35±0.88, 42.39±23.8, and 8.30±37.1 days per 30 months, respectively (Table 1).

**Table 1:** Baseline and work characteristics of study population (304 healthcare providers).

Mean age, year	35.00 ± 8.38
Gender, female	247 (81.3%)
Marital status, married	167 (55.0%)
Education level	
Master	
Bachelor	
Under Bachelor	35 (11.6%)
235 (77.6%)	
33 (10.8%)	
Having child	114 (37.5%)
Underlying disease	48 (15.8%)
Using different drugs	18 (5.9%)
Smoking	48 (15.8%)
Mean (SD) number of children	0.58 ± 0.85
Mean (SD) job experience, year	10.01 ± 7.14
Mean (SD) overtime work, hour	82.01 ± 43.88
Mean (SD) body mass index, kg/m^2^	24.82 ± 3.91
Mean (SD) systolic blood pressure, mmHg	111.16 ± 11.49
Mean (SD) diastolic blood pressure, mmHg	69.30 ± 9.00
Mean (SD) distance from work place, km	25.82 ± 27.38
Task	
Head nurse	12 (4.1%)
Nurse	243 (82.4%)
Paramedic	33 (11.2%)
Operating room technician	7 (2.3%)
Workplace (ward)	
Internal	61 (21.2%)
Surgery	76 (26.5%)
CCU & ICU & NICU	64 (22.3%)
Others	86 (30%)
Having shift work	273 (89.8%)
Types of shifts:	
Only morning	31 (10.6%)
Only night	24 (8.1%)
Morning/evening	78 (26.4%)
Morning/evening/night	136 (46.1%)
Evening/night	26 (8.8%)
Having second job	37 (12.2%)
Having third job	2 (0.7%)
Mean (SD) time for unauthorized absence, days	0.35 ± 0.88
Mean (SD) time for vacation, days	42.39 ± 23.80
Mean (SD) time for sick absence, days	8.30 ± 3.11

Overall, 46.1% went to bed at midnight. Real sleep hours less than 5 hours were expressed by 19.4%. The use of sedatives or hypnotics for sleeping was once per week in 6.6%, twice weekly in 2.3% and three times or more per week in 2.6%. Regarding difficulties in sleeping, severe to very severe impairment in falling asleep, staying asleep, and in waking up very early was expressed by 9.6%, 10.9%, and 13.1%, respectively. Also, 21.0% of the nursing team were dissatisfied or had very little satisfaction. 53% percent believed that sleep difficulty interferes severely with their daily activities. In addition, 29.0% also found that quality of life could be disturbed by sleep difficulties. Also, 23.4% were very worried about their sleep (Table 2).

**Table 2:** Difficulties in sleeping among 304 healthcare providers according to scores of 7 items of insomnia severity index.

ISI items score	0	1	2	3	4
1 Hard to fall asleep^1^ N (%)	100 (32.9)	101 (33.2)	74 (24.3)	26 (8.6)	3 (1.0)
2 Hard to staying asleep^1^ N (%)	103 (33.9)	97 (31.9)	71 (23.4)	26 (8.6)	7 (2.2)
3 Waking up very early^1^ N (%)	111 (36.5)	82 (27.0)	71 (23.4)	28 (9.2)	12 (3.9)
4 Sleep satisfaction^2^ N (%)	32 (10.6)	50 (16.4)	158 (52.0)	50 (16.4)	14 (4.6)
5 Interfering with daily activities^2^ N (%)	27 (8.8)	34 (11.2)	82 (27.0)	92 (30.3)	69 (22.7)
6 Interfering with quality of life^2^ N (%)	37 (12.1)	72 (23.7)	107 (35.2)	54 (17.8)	34 (11.2)
7 Worry about sleep status^2^ N (%)	69 (22.7)	77 (25.3)	87 (28.6)	55 (18.1)	16 (5.3)

^1^ The score of 0, 1, 2, 3, 4 indicating non, mild, moderate, severe, and very severe, respectively;

^2^ The score of 0, 1, 2, 3, 4 not at all, a little, somewhat, much, and very much, respectively.

Overall, the mean ISI score was 11.26±4.26 in total, different degrees of insomnia was found in 79.9% of the study population that 57.2% suffered from subthreshold insomnia (8-14), 21.4% from moderate insomnia (15-21), and 1.3% from severe insomnia (22-28). With regard to the relationship between the severity of insomnia based on the ISI score and baseline variables, women had higher ISI score compared to men (11.60±4.24 vs. 9.88±4.10, *p*=0.006); but, the severity of insomnia was independent to other parameters including age, marital status, education level, body mass index, overtime working, workplace (wards of hospitals), the presence of second or third job, underlying disease or use of medications, smoking, and distance between home to workplace. There was no association between the type of work shift and the presence of insomnia (*p*=0.19).

The prevalence of insomnia was significantly higher in the healthcare providers who were absent from their workplace frequently, or left there because of illness. The *p* for trend was used to test a linear trend of absenteeism rate between 3 categories of insomnia grading (no insomnia, mild insomnia, and moderate to severe insomnia). In this regard, the mean days for unauthorized absenteeism in persons with no, mild, moderate to severe insomnia was 0.16±0.45, 0.37±0.87, and 0.49±1.13 days, respectively (*p*-value trend=0.03), the mean time for absenteeism due to illness was also 1.70±5.04, 6.94±32.8, and 17.59±56.96, respectively (*p-*value trend=0.01) and the mean time for total absenteeism was 43.3±17.5, 49.8±44.6, 60.9±62.14, respectively (*p*-value trend=0.03) (Table 3).

**Table 3:** The unauthorized, vacation and sick absenteeism rates in study population and association between absenteeism and insomnia index.

Insomnia severity index	Unauthorized absenteeism^1^ Mean (SD)	Vacation absenteeism^1^ Mean (SD)	Sick absenteeism^1^ Mean (SD)	Vacation and sick absenteeism^1^ Mean (SD)	Total absenteeism^1^ Mean (SD)
Total (N=304)	0.35±.88	42.39±23.80	8.31±37.11	50.70±45.74	51.05±45.86
No insomnia (N=61)	0.16±0.45	41.52±17.31	1.70±5.04	43.23±17.59	43.39±17.58
Mild insomnia (N=174)	0.37±0.87	42.51±25.63	6.94±32.82	49.44±44.66	49.81±44.69
Moderate to severe insomnia (N=69)	0.49±1.13	42.87±24.23	17.59±56.96	60.46±61.84	60.96±62.14
*p*-value trend	0.03	0.75	0.01	0.03	0.03

^1^ Days in period of 30 months.

Then we used regression analysis to examine if insomnia grading is predictor of absenteeism in nursing team. In a multivariable linear regression analysis, of all baseline variables, gender, number of children, having underlying diseases, and shift type could predict insomnia. On the other side, gender, having second job, having night shifts, and severity of insomnia could predict absenteeism in studied nursing team (Table 4).

**Table 4:** The multivariable linear regression model for predicting determinants of insomnia and absenteeism among nurses.

Variable in model	ISI Score		Total absenteeism	
	B	Sig	B	Sig
Constant	-	0.21	-	0.96
Age	- 0.63	0.29	0.31	0.56
Gender	0.43	0.04	-0.63	0.02
Number of children	0.44	0.03	-0.24	0.25
Age of first child	-0.48	0.25	-0.60	0.18
Age of second child	1.03	0.09	0.34	0.56
Smoking	0.25	0.38	-0.19	0.47
Having underlying disease	0.64	0.009	-0.15	0.59
Having Second job	0.32	0.15	-0.48	0.04
Task^1^	-0.20	0.48	0.02	0.93
Over time work	0.04	0.87	0.28	0.24
Having shift work	0.70	0.11	0.85	0.08
Shift type^2^	-1.25	0.04	-1.29	0.06
Night shift	-0.20	0.40	0.93	0.004
Insomnia grading	-	-	0.80	0.02
Model R square	0.56		0.63	

^1^ Tasks including: nurses, head nurses, paramedics and operating room technician;

^2^ Shift type: only morning, only night, morning & evening, morning & evening & night, evening & night.

## DISCUSSION

Long-term absence from work or repeated absenteeism is a serious risk profile for permanently leaving the job, which leads to the loss of an efficient and experienced workforce. Proper job perfor mance is the result of a combination of physical and mental health factors and it is obvious that with mastery of different underlying physical and mental disturbances, the likelihood of permanent absenteeism can be increased. In this regard, it is now suggested that insomnia (that is on its own the result of the high workload and poor quality of the community health management system) is closely linked to absenteeism among healthcare providers. Furthermore, in this study, absenteeism among nursing team is strongly associated with the severity of insomnia even after adjusting baseline demographic and work-related factors. In other words, high workload among the nursing team can lead to sleep disorders. Having feeling of sleepiness and discomfort in work environment reduces the quality of service delivering to patients and increases risk of occupational errors occurrence. Moreover, it leads to a tendency for long vacations, even temporary and sometimes leaving the workplace permanently. Of course, it should be noted that the population of nursing team who included in this study work in a referral hospital in Tehran and all of them lived in Tehran, which is known as one of the busiest cities in the world. Ultimately, the triangle of workload, the living environment, and sleep disorders will all lead to nursing teams’ absenteeism and ultimately the desire to leave the workplace.

As indicated similarly by Lamont et al. (2017)^20^, 44% of the nurse and midwife respondents took sickness absence and in this regard, those affected were significantly more likely to be at younger ages, working shifts with less time sitting at work, to report workplace abuse and plans to leave, to be current smokers, to report mental health problems, accomplishing less due to emotional problems, and current psychotropic medication use.

The physiological function and rhythm of the body’s circulatory system changes by working in hours outside the designed window (between 6 and 18 hour), which disrupts the secretion of body hormones including cortisol and aldosterone enzymes and ultimately leads to sleep disorders and other adverse health effects in shift workers^21^. In addition, working on shifts results in feeling of moodiness by inducing a decrease in serotonin, a substance that regulates sleep, promotes good mood, and is released during the night^22^. As shown by Chiang et al. (2012)^23^, working tenure has also been found to be a significant predictor of stress levels, depression, and intention to leave nursing. Portela et al. (2015)^24^ showed that the prevalence of insomnia symptoms was 34.3% and job strain was associated with increased odds for insomnia symptoms; the same result was observed with the combination of emotional demands and low job control. In a similar study by Hui et al. (2015)^25^, those nurses who had higher levels of sleep disturbance were more likely to be absent from work, have lower work performance ratings, and have higher healthcare costs and thus as similarly concluded in our study, more trouble sleeping was significantly related to negative changes in longer absence from work. Bültmann et al. (2013)^26^ found that sleep disturbances and fatigue significantly predicted sickness absences. Rahkonen et al. (2012)^27^ also found that frequent sleep problems were associated with increased sickness absences, both short and long in duration.

Findings of this study revealed there is a linear trend of absenteeism rate including unauthorized absenteeism, sick absenteeism and total absenteeism between 4 categories of insomnia grading (*p*-value for trend <0.05). Therefor by developing screening programs, subthreshold insomnia among nursing team can be identified. Then implementation of some effective interventions such as education of sleep hygiene principles and shift scheduling modifications can prevent from adverse outcomes of moderate and severe insomnia.

In total, according to the literatures, it seems that the employees’ sleep disturbances can be associated with a wide variety of negative occupational outcomes such as absenteeism and decreased productivity. The absence of nurses results in staff shortages and an additional workload on their colleagues. In addition, absenteeism can be a significant predictor of future and long-term absenteeism and permanent work leave^28, 29^. This issue will be clearly detrimental to the organization of hospitals and the quality of patients care in long term. Employee turnover imposes higher costs to the hospital organization including recruiting and training expenses and some hidden costs such as productivity loss, workplace safety issues. Therefore, managerial discussion on absenteeism is clearly warranted.

## CONCLUSION

In conclusion, a majority of nursing team suffer from insomnia that may lead to their reluctance to continue their continuous and quality work activities, which also leads to long vacations, sick leaves, or even a full-time job leaving. Considering the importance of this subject, it is worth focusing on shift scheduling modifications education of sleep hygiene principles to help alleviate the burden of insomnia among nursing team.
